# Does surfactant nebulization prevent early intubation in preterm infants? A protocol for a systematic review and meta-analysis

**DOI:** 10.1186/s13643-020-01573-6

**Published:** 2021-01-06

**Authors:** Vincent D. Gaertner, Dirk Bassler, Christoph M. Rüegger

**Affiliations:** Newborn Research, Department of Neonatology, University Hospital and University of Zurich, Zurich, Switzerland

**Keywords:** Nebulized surfactant, Delivery room, Respiratory distress syndrome, Bronchopulmonary dysplasia, Aerosolized surfactant, Surfactant nebulization, Surfactant aerosolization, Meta-analysis

## Abstract

**Background:**

Respiratory distress syndrome (RDS) is the most common cause of respiratory failure in preterm infants. Treatment consists of respiratory support and exogenous surfactant administration. Commonly, surfactant is administered intratracheally. However, this requires airway instrumentation and subsequent fluid instillation which may be harmful. Surfactant nebulization (SN) may offer a safe and effective alternative for surfactant administration, but the clinical efficacy is not yet established. Thus, this systematic review and meta-analysis of randomized controlled trials will summarize the available evidence to determine the effectiveness and safety of SN for the prevention of intubation and subsequent mechanical ventilation at 72 h after birth.

**Methods:**

A systematic literature search in Medline, Embase, and The Cochrane Library will be performed, and all randomized controlled trials (RCTs) and quasi-RCTs from published articles, presentations, and trial registries will be included in this meta-analysis. Titles and abstracts of all records identified in the search will be screened by two reviewers independently.

Data on preterm infants (≤ 37 weeks) receiving nebulized surfactant in the first 72 h after birth for the treatment or prevention of RDS will be evaluated. Primary outcome is the intubation rate by 72 h after birth, and secondary outcomes include peridosing safety effects as well as major neonatal morbidities.

Risk of bias will be assessed using the revised Cochrane ROB tool, and subgroup analyses will be performed to evaluate potential confounding factors. Publication bias will be assessed by examining a funnel plot. The meta-analysis will be performed using a fixed-effects model.

**Discussion:**

This review will provide an evidence-based tool for information about surfactant nebulization, illustrating the current knowledge and hopefully revealing potential novel avenues for researchers and clinicians alike.

**Systematic review registration:**

This review is registered with the publicly available resource PROSPERO (CRD42020175625).

**Supplementary Information:**

The online version contains supplementary material available at 10.1186/s13643-020-01573-6.

## Background

### Clinical background

Depending on the level of prematurity, approximately 50–90% of very preterm infants with a gestational age below 32 weeks at birth suffer from respiratory distress syndrome (RDS) [1, 2]. Respiratory distress syndrome is the most common cause of respiratory failure in preterm infants. It is related to a primary surfactant deficiency leading to an unphysiologically high pulmonary resistance, increased work of breathing, and ultimately, higher pressures needed for mechanical ventilation [1]. This in turn can lead to epithelial lesions, the formation of hyaline membranes, increased inflammation and ultimately to the development of bronchopulmonary dysplasia (BPD) [3, 4].

Treatment consists of respiratory support and exogenous surfactant administration. Current neonatal resuscitation guidelines advise to stabilize preterm infants on continuous positive airway pressure (CPAP) initially and administer surfactant as early rescue therapy < 2 h after birth, but only if respiratory compromise is suspected [5, 6]. While the benefit of surfactant has been widely investigated, the best route of surfactant application is not yet determined [7–11]. Surfactant is commonly instilled into the trachea, either by endotracheal intubation and subsequent mechanical ventilation or by inserting a nasogastric tube into the trachea and removing it immediately after instillation (MIST, minimally invasive surfactant therapy). MIST may reduce volutrauma and airway inflammation [12–14]. However, there is still the need for instrumentation of the airway and fluid instillation into the trachea. Instrumentation of the airway is uncomfortable and painful for the preterm infant and may carry the risk of concomitant vasovagal reactions and pharyngeal/laryngeal injury. Furthermore, surfactant instillation into the trachea leads to disruption of the cerebral blood flow and mean arterial blood pressure [15]. Additionally, intratracheal surfactant administration and mechanical ventilation are associated with the development of intraventricular hemorrhage [16–18].

In recent years, there has been an increasing shift towards less invasive treatment options for RDS in preterm infants. Thus, a truly noninvasive approach for surfactant administration which avoids clinical instability and allows for repeated surfactant applications is still needed. Surfactant nebulization (SN) may offer such an alternative as it may be helpful in avoiding the need for mechanical ventilation or instrumentation of the upper airways [19].

### Animal data

There is various animal data supporting the application of SN: Nebulized surfactant improved lung compliance and oxygenation in preterm lambs when compared to saline nebulization [20]. In rabbits, surfactant nebulization led to a more homogeneous surfactant distribution when compared to instillation [21]. Further studies showed reduced inflammatory markers when comparing SN + CPAP to CPAP alone and demonstrated reduced adverse hemodynamic effects of SN compared with intratracheal surfactant instillation [15, 22–27]. Furthermore, SN has been shown to improve cerebral oxygen metabolism when compared with intratracheal surfactant bolus administration [27]. The clinical efficacy of SN in animal studies was similar to intratracheal surfactant application in improving the oxygenation index, the arterial oxygen and carbon dioxide partial pressure as well as the ventilation efficacy index [28].

### Clinical data

While animal data show promising effects of SN, clinical results to date are ambiguous and scarce [19]. There is no single large trial evaluating the efficacy of surfactant nebulization but various smaller studies with varying results [29–33]. Surfactant nebulization was associated with a drop in partial carbon dioxide pressure [29] and with a decreased required inspiratory fraction of oxygen [32]. In other studies, there was no clinical benefit for infants receiving SN [30, 31]. Therefore, this systematic review and meta-analysis will assess the efficacy of SN by combining studies of high methodological rigor and will potentially reveal relevant factors associated with a higher or lower efficacy.

### Research question

This systematic review and meta-analysis of randomized controlled trials will aim to evaluate the effectiveness of SN in preterm infants with RDS. More specifically, this review tries to answer the question whether surfactant nebulization is effective in decreasing intubation rate by 72 h after birth in preterm infants with RDS (≤ 37 weeks gestational age), when compared with standard care or other forms of surfactant application.

## Methods

The present protocol has been registered in the PROSPERO database (registration number (CRD42020175625) and is being reported in accordance with the reporting guidance provided in the Preferred Reporting Items for Systematic Reviews and Meta-Analyses Protocols (PRISMA-P) statement [34] (see checklist in Additional file 1).

### Searches

A systematic literature search will be conducted on the following electronic databases: Medline, Embase, and The Cochrane Library [Cochrane Database of Systematic Reviews, Cochrane Central Register of Controlled Trials (CENTRAL)]. In addition, we will seek registered details of selected trials in the US National Institutes of Health resource ClinicalTrials.gov. We will obtain information by personal communication with the corresponding authors and will review the reference lists of relevant articles, abstracts, and conference proceedings (Pediatric Academic Societies, Society for Pediatric Research, European Society for Pediatric Research 1990–2019). There will be no language restrictions. The searches will be re-run just before the final analyses and further studies retrieved for inclusion.

We will search the electronic databases mentioned above using stringent search terms, e.g., the following for EMBASE:

((‘prematurity’/exp OR ‘infant’/exp OR prematur*:ti,ab OR preterm*:ti,ab OR newborn*:ti,ab ORinfant*:ti,ab OR neonat*:ti,ab) AND (‘lung surfactant’/exp OR ((surfactant NEAR/5 (nebuli* ORaerosoli* OR administ*)):ti,ab)) NOT ([animals]/lim NOT [humans]/lim)) AND (‘crossover procedure’:de OR ‘double-blind procedure’:de OR 'randomized controlled trial’:de OR’single-blind procedure’:de OR random*:de,ab,ti OR factorial*:de,ab,ti OR crossover*:de,ab,ti OR((cross NEXT/1 over*):de,ab,ti) OR placebo*:de,ab,ti OR ((doubl* NEAR/1 blind*):de,ab,ti) OR((singl* NEAR/1 blind*):de,ab,ti) OR assign*:de,ab,ti OR allocat*:de,ab,ti OR volunteer*:de,ab,ti)

### Types of study to be included

We will include all randomized controlled trials (RCTs) and quasi-RCTs. We will take into account the level at which randomization occurred, including cross-over trials, cluster-randomized trials, and multiple observations for the same outcome. All animal studies will be excluded from analysis. We will extract relevant data published in journals as well as data only available in online clinical trial registries to perform a meta-analysis and evaluate whether there is a significant benefit of nebulized surfactant in preventing intubation and subsequent mechanical ventilation at 72 h after birth.

### Condition or domain being studied

This systematic review will summarize the available evidence from randomized controlled trials to determine the effectiveness and safety of SN for the prevention of intubation and subsequent mechanical ventilation at 72 h after birth.

### PICO: participants–intervention–comparator–outcome

Participants: Preterm infants (≤ 37 weeks) with RDS in the first 72 h after birth

Intervention: Nebulized surfactant administration for the prevention or treatment of RDS during the first 72 h after birth

Comparator/control: Any control group will be included. Thus, surfactant nebulization could be compared to no treatment, to CPAP alone, or to a more invasive surfactant administration method.

Outcomes:

- Primary outcome: intubation rate by 72 h after birth
- Secondary outcomes by timepoint:
  - Peridosing events:
    - Number of bradycardias ≤ 80 bpm during nebulization measured by electrocardiography or pulse oximetry
    - Number of desaturations ≤ 80% FiO_2_ during nebulization measured by pulse oximetry
    - Vomiting during nebulization
  - During or within the first 72 h after SN:
    - Oxygen saturation and arterial carbon dioxide levels at 1, 2, and 3 h after SN when compared with baseline at the beginning of SN
    - SpO_2_/FiO_2_ ratio at 1 h, as well as at 24, 48, and 72 h after SN when compared with baseline at the beginning of SN
    - Mean airway pressure level at 1 h, as well as at 24, 48, and 72 h after SN when compared with baseline at the beginning of SN
    - Electrolyte imbalances (e.g., increase/decrease in sodium, potassium, calcium, or chloride levels 24 h after SN when compared with baseline at the beginning of SN)
  - Until 36 weeks postmenstrual age
    - Blood-culture positive neonatal sepsis before 36 weeks postmenstrual age
    - Pneumothorax or pulmonary interstitial emphysema
    - Severe intraventricular hemorrhage (grade 3 or 4 based on the Papile criteria [35])
    - Any stage retinopathy of prematurity based on the International Classification of Retinopathy of Prematurity (2005) [36]
    - Necrotizing enterocolitis (stage 2 or higher according to the Bell classification) [37]
    - Pulmonary hemorrhage
  - At 36 weeks postmenstrual age:
    - Number of days on mechanical ventilation during hospitalization
    - Intubation rate during hospitalization (i.e. number of infants who were intubated at any time during hospitalization)
    - Death
    - Moderate to severe BPD (defined as oxygen requirement at 36 weeks postmenstrual age)
  - At 18–24 months corrected age:
    - Neurodevelopment, i.e., number of infants with a Bayley-III composite motor or cognitive score of more than two SD below the mean (Bayley Scales of Infant and Toddler Development, third edition [38])

### Study selection and data extraction

Two reviewers will independently screen the titles and abstracts of all records identified in the search and obtain full texts of the relevant trials. The full texts will be assessed, and those studies that do not meet all of the inclusion criteria will be excluded. Any disagreements will be discussed until consensus is achieved, if necessary through referral to a third reviewer. Records of ineligible full-text articles along with the reason for ineligibility will be saved for future reference. We will contact authors of primary studies to provide any missing information every month for three consecutive months.

### Risk of bias (quality) assessment

The risk of bias (ROB) of eligible studies will be assessed according to the Revised Cochrane risk-of-bias tool for randomized trials (RoB 2) [39]. The six criteria to be assessed are sequence generation, allocation concealment, blinding of participants, personnel and outcome assessors, completeness of follow-up, selective outcome reporting, and presence of other biases. Each domain will be assigned a score “definitely low risk” or “definitely high risk” or “unclear risk”. We will further categorize the “unclear risk” into “probably low risk” or “probably high risk”. The confidence in the estimates for each outcome will be assessed using the GRADE (Grading of Recommendations Assessment, Development and Evaluation) approach. We will assess reporting and publication bias by examining the degree of asymmetry of a funnel plot.

### Heterogeneity and subgroup analysis

We propose the following potential sources of clinical heterogeneity, which could be possible effect modifiers: gestational age, birth weight, surfactant dose, timing of surfactant, type of nebulizer, type of surfactant, and frequency of nebulization. We hypothesize that a lower gestational age and a lower birth weight will be related to a better effectiveness of SN. Furthermore, we speculate that a higher surfactant dose and more frequent applications lead to a better outcome and that different types of nebulizer show different effectiveness. Additionally, there are potential methodological sources of heterogeneity such as risk of bias, study design, publication bias, or type of randomization. Sensitivity analysis will be conducted including only the high-quality studies (with “low” or “probably low” risk of bias) if possible.

Meta-regression models will assume a single shared coefficient for all non-baseline treatments [40]. Interpretation of meta-regression models will be in keeping with recent suggestions, namely, inclusion of the coefficient leads to a decrease in the estimate of between-study variance [40]. A meta-regression will be conducted exploring the effect of the proposed sources of heterogeneity on the effectiveness of SN depending on availability of data.

### Strategy for data synthesis and measures of treatment effect

Data will be analyzed as aggregate. Effect estimates along with 95% compatibility intervals (CIs) will be primarily presented using risk ratios (RRs). Odds ratios (ORs) will be reported as a supplementary analysis to account for potential distortion of the RRs. The meta-analysis will be performed using a fixed-effects model.

## Discussion

This systematic review and meta-analysis will summarize the available evidence from randomized controlled trials to determine the safety and effectiveness of SN for the prevention of intubation and subsequent mechanical ventilation at 72 h after birth.

The last Cochrane review on the effectiveness of SN was performed in 2012 and included a single randomized controlled trial [41]. There have been further reviews of clinical data over the past years [19, 23, 42]. However, a recent randomized controlled trial examining the effectiveness of SN was not included so far [33], and several more studies with a higher sample size are about to be published (e.g., NCT03058666, NCT03235986). Also, all the aforementioned reviews included only studies published in journals while disregarding studies where results are only available in online trial registries. The results of this systematic review and meta-analysis will be of interest to a broad range of neonatologists, pediatricians, and researchers. Most importantly, it will show avenues of further research and possible clinical applications to further improve noninvasive RDS therapy in preterm infants which is of utmost importance in preventing associated long-term impairments.

Our review will have several strengths. First, we will implement a wide comprehensive search to include published as well as thus far unpublished clinical trials. Second, we will provide all-encompassing information on all studies currently available and thus increase the informative value of this review for clinicians and researchers. Third, we will primarily focus on clinically relevant outcomes such as intubation and mechanical ventilation which are among the major risk factors associated with the development of BPD. On the other hand, the chosen primary outcome is a short-term clinical outcome while long-term neurodevelopmental outcomes may be more important to patients, parents, and caregivers. However, we are not aware of any trial assessing the efficacy of SN in reducing death and/or major neurodevelopmental impairment. Also, it is known that intubation in general as well as the duration of mechanical ventilation is associated with the development of BPD [43], while BPD itself is associated with a worse neurodevelopmental outcome [44]. Thus, our approach may yield clinically relevant results which may provide the rationale and background information to conduct a larger trial focusing on long-term outcomes. Also, we included death and major neonatal morbidities as secondary outcomes to assess whether the smaller trials that have been conducted to date may show a combined impact on any of the longer-term outcomes.

We anticipate some challenges while undertaking such a review. Over the last years, many new devices for surfactant nebulization have been conceived and built in order to increase the amount of surfactant reaching the lower airways, thereby aiming to improve clinical efficacy [45–47]. Thus, we anticipate some degree of clinical heterogeneity as we are considering different devices but also different timepoints of application and a wide range of gestational ages.

To summarize, we hope that this review will provide information about surfactant nebulization with a high level of evidence, illustrating the current knowledge and hopefully revealing potential novel avenues for researchers and clinicians alike. The findings of this review may lead to the design of adequately powered trials assessing longer-term outcomes and ultimately to an improved medical care of this vulnerable population.

## Supplementary Information

**Additional file 1.** PRISMA-P 2015 Checklist.

## Data Availability

The datasets used and/or analyzed during the current study are available from the corresponding author on reasonable request.

## Abbreviations

BPD: Bronchopulmonary dysplasia
CPAP: Continuous positive airway pressure
GRADE: Grading of Recommendations Assessment, Development, and Evaluation
MIST: Minimally invasive surfactant therapy
PRISMA-P: Preferred Reporting Items for Systematic Reviews and Meta-Analyses Protocols
RCT: Randomized controlled trial
RDS: Respiratory distress syndrome
ROB: Risk of bias
SN: Surfactant nebulization

## References

[1] Sweet DG, Carnielli V, Greisen G, Hallman M, Ozek E, Plavka R (2013). European consensus guidelines on the management of neonatal respiratory distress syndrome in preterm infants--2013 update. Neonatology..

[2] EuroNeoStat (2010). Annual report for very low gestational age infants 2010.

[3] Joshi S, Kotecha S (2007). Lung growth and development. Early Hum Dev..

[4] Smith LJ, McKay KO, van Asperen PP, Selvadurai H, Fitzgerald DA (2010). Normal development of the lung and premature birth. Paediatr Respir Rev..

[5] Sweet DG, Carnielli V, Greisen G, Hallman M, Ozek E, Plavka R (2017). European consensus guidelines on the management of respiratory distress syndrome - 2016 Update. Neonatology..

[6] Polin RA, Carlo WA, Committee on Fetus and Newborn, American Academy of Pediatrics (2014). Surfactant replacement therapy for preterm and term neonates with respiratory distress. Pediatrics..

[7] Finer NN, Carlo WA, Walsh MC, Rich W, Gantz MG, SUPPORT Study Group of the Eunice Kennedy Shriver NICHD Neonatal Research Network SSG of the EKSNNR (2010). Early CPAP versus surfactant in extremely preterm infants. N Engl J Med.

[8] Morley CJ, Davis PG, Doyle LW, Brion LP, Hascoet J-M, Carlin JB (2008). Nasal CPAP or intubation at birth for very preterm infants. N Engl J Med..

[9] Dunn MS, Kaempf J, de Klerk A, de Klerk R, Reilly M, Howard D (2011). Randomized trial comparing 3 approaches to the initial respiratory management of preterm neonates. Pediatrics..

[10] Niemarkt HJ, Hütten MC, Kramer BW (2017). Surfactant for respiratory distress syndrome: new ideas on a familiar drug with innovative applications. Neonatology..

[11] Blennow M, Bohlin K (2015). Surfactant and noninvasive ventilation. Neonatology..

[12] Kanmaz HG, Erdeve O, Canpolat FE, Mutlu B, Dilmen U (2013). Surfactant administration via thin catheter during spontaneous breathing: randomized controlled trial. Pediatrics..

[13] Isayama T, Iwami H, McDonald S, Beyene J (2016). Association of noninvasive ventilation strategies with mortality and bronchopulmonary dysplasia among preterm infants. JAMA..

[14] Göpel W, Kribs A, Härtel C, Avenarius S, Teig N, Groneck P (2015). Less invasive surfactant administration is associated with improved pulmonary outcomes in spontaneously breathing preterm infants. Acta Paediatr..

[15] Dijk PH, Heikamp A, Bambang Oetomo S (1997). Surfactant nebulisation prevents the adverse effects of surfactant therapy on blood pressure and cerebral blood flow in rabbits with severe respiratory failure. Intensive Care Med..

[16] Klebermass-Schrehof K, Wald M, Schwindt J, Grill A, Prusa A-R, Haiden N (2013). Less invasive surfactant administration in extremely preterm infants: impact on mortality and morbidity. Neonatology..

[17] Helwich E, Rutkowska M, Bokiniec R, Gulczyńska E, Hożejowski R (2017). Intraventricular hemorrhage in premature infants with Respiratory Distress Syndrometreated with surfactant: incidence and risk factors in the prospective cohort study. Dev Period Med..

[18] Nayeri FS, Esmaeilnia Shirvani T, Aminnezhad M, Amini E, Dalili H, Moghimpour Bijani F (2014). Comparison of INSURE method with conventional mechanical ventilation after surfactant administration in preterm infants with respiratory distress syndrome: therapeutic challenge. Acta Med Iran..

[19] Pillow JJ, Minocchieri S (2012). Innovation in surfactant therapy II: surfactant administration by aerosolization. Neonatology..

[20] Lewis JF, Ikegami M, Jobe AH, Tabor B (1991). Aerosolized surfactant treatment of preterm lambs. J Appl Physiol..

[21] Schermuly R, Schmehl T, Günther A, Grimminger F, Seeger W, Walmrath D (1997). Ultrasonic nebulization for efficient delivery of surfactant in a model of acute lung injury. Impact on gas exchange. Am J Respir Crit Care Med..

[22] Hütten MC, Kuypers E, Ophelders DR, Nikiforou M, Jellema RK, Niemarkt HJ (2015). Nebulization of poractant alfa via a vibrating membrane nebulizer in spontaneously breathing preterm lambs with binasal continuous positive pressure ventilation. Pediatr Res..

[23] Walther FJ, Hernández-Juviel JM, Waring AJ (2014). Aerosol delivery of synthetic lung surfactant. PeerJ..

[24] Wolfson MR, Wu J, Hubert TL, Mazela J, Gregory TJ, Clayton RG (2011). Dose-response to aerosolized KL4 surfactant in the spontaneously breathing CPAP-supported preterm lamb. Pediatr Res..

[25] Milesi I, Tingay DG, Lavizzari A, Bianco F, Zannin E, Tagliabue P (2017). Supraglottic atomization of surfactant in spontaneously breathing lambs receiving continuous positive airway pressure. Pediatr Crit Care Med..

[26] Milesi I, Tingay DG, Zannin E, Bianco F, Tagliabue P, Mosca F (2016). Intratracheal atomized surfactant provides similar outcomes as bolus surfactant in preterm lambs with respiratory distress syndrome. Pediatr Res..

[27] Rey-Santano C, Mielgo VE, López-de-Heredia-y-Goya J, Murgia X, Valls-i-Soler A (2016). Cerebral effect of intratracheal aerosolized surfactant versus bolus therapy in preterm lambs. Crit Care Med..

[28] Bianco F, Ricci F, Catozzi C, Murgia X, Schlun M, Bucholski A (2019). From bench to bedside: in vitro and in vivo evaluation of a neonate-focused nebulized surfactant delivery strategy. Respir Res..

[29] Jorch G, Hartl H, Roth B, Kribs A, Gortner L, Schaible T (1997). Surfactant aerosol treatment of respiratory distress syndrome in spontaneously breathing premature infants. Pediatr Pulmonol..

[30] Arroe M, Pedersen-Bjergaard L, Albertsen P, Bodé S, Greisen G, Jonsbo F (1998). Inhalation of aerosolized surfactant (exosurf) to neonates treated with nasal continuous positive airway pressure. Prenat Neonat Med..

[31] Berggren E, Liljedahl M, Winbladh B, Andreasson B, Curstedt T, Robertson B (2000). Pilot study of nebulized surfactant therapy for neonatal respiratory distress syndrome. Acta Paediatr..

[32] Finer NN, Merritt TA, Bernstein G, Job L, Mazela J, Segal R (2010). An open label, pilot study of Aerosurf® combined with nCPAP to prevent RDS in preterm neonates. J Aerosol Med Pulm Drug Deliv..

[33] Minocchieri S, Berry CA, Pillow JJ, CureNeb Study T (2019). Nebulised surfactant to reduce severity of respiratory distress: a blinded, parallel, randomised controlled trial. Arch Dis Child Fetal Neonatal Ed..

[34] Moher D, Shamseer L, Clarke M, Ghersi D, Liberati A, Petticrew M, Shekelle P, Stewart LA, PRISMA-P Group. Preferred reporting items for systematic review and meta-analysis protocols (PRISMA-P) 2015 statement. Syst Rev. 2015;4(1):1. 10.1186/2046-4053-4-1. 10.1186/2046-4053-4-1 PMC4320440 25554246

[35] Papile LA, Burstein J, Burstein R, Koffler H (1978). Incidence and evolution of subependymal and intraventricular hemorrhage: a study of infants with birth weights less than 1,500 gm. J Pediatr..

[36] International Committee for the Classification of Retinopathy of Prematurity (2005). The International Classification of Retinopathy of Prematurity revisited. Arch Ophthalmol (Chicago, Ill 1960).

[37] Bell MJ, Ternberg JL, Feigin RD, Keating JP, Marshall R, Barton L (1978). Neonatal necrotizing enterocolitis. Ann Surg..

[38] Bayley N (2006). Bayley scales of infant and toddler development, third edition: Administration manual.

[39] Sterne JAC, Savović J, Page MJ, Elbers RG, Blencowe NS, Boutron I (2019). RoB 2: a revised tool for assessing risk of bias in randomised trials. BMJ..

[40] Dias S, Sutton AJ, Welton NJ, Ades AE (2013). Evidence synthesis for decision making 3. Med Decis Mak..

[41] Abdel-Latif ME, Osborn DA. Nebulised surfactant in preterm infants with or at risk of respiratory distress syndrome. Cochrane Database Syst Rev. 2012;10:CD008310. 10.1002/14651858.CD008310.pub2 PMC11800327 23076945

[42] More K, Sakhuja P, Shah PS (2014). Minimally invasive surfactant administration in preterm infants. JAMA Pediatr..

[43] Rutkowska M, Hożejowski R, Helwich E, Borszewska-Kornacka MK, Gadzinowski J (2019). Severe bronchopulmonary dysplasia - incidence and predictive factors in a prospective, multicenter study in very preterm infants with respiratory distress syndrome. J Matern neonatal Med..

[44] Schmidt B, Asztalos EV, Roberts RS, Robertson CMT, Sauve RS, Whitfield MF (2003). Impact of bronchopulmonary dysplasia, brain injury, and severe retinopathy on the outcome of extremely low-birth-weight infants at 18 months: results from the trial of indomethacin prophylaxis in preterms. JAMA..

[45] Fink JB, Macloughlin R, Telfer C, Clark A (2016). Developing inhaled drugs for critically ill patients: a platform for all ages.

[46] Minocchieri S, Knoch S, Schoel WM, Ochs M, Nelle M (2014). Nebulizing poractant alfa versus conventional instillation: ultrastructural appearance and preservation of surface activity. Pediatr Pulmonol..

[47] Pohlmann G, Iwatschenko P, Koch W, Windt H, Rast M, de Abreu MG (2013). A novel continuous powder aerosolizer (CPA) for inhalative administration of highly concentrated recombinant surfactant protein-C (rSP-C) surfactant to preterm neonates. J Aerosol Med Pulm Drug Deliv..

